# Comparative outcomes of arthroscopy-assisted uniportal spinal surgery vs. unilateral biportal endoscopy for adjacent two-segment lumbar disc herniation: a retrospective cohort study

**DOI:** 10.3389/fsurg.2026.1825301

**Published:** 2026-05-22

**Authors:** Cheng He, Hao Li, Haoli Zhang, Zhengping Zhang, Zhilin Li, En Song, Xigao Cheng, Jiangjun Zhou

**Affiliations:** 1 Department of Orthopedic, The 908th Hospital of Chinese People’s Liberation Army Joint Logistic Support Force, Nanchang, Jiangxi, China; 2 Department of Spine Surgery, Xi’an Jiaotong University Affiliated Honghui Hospital, Xi'an, Shaanxi, China; 3 Department of Orthopedic, Guangyuan Central Hospital, Guangyuan, Sichuan, China; 4 Department of Sports Medicine, The First Affiliated Hospital of Kunming Medical University, Kunming, Yunnan, China; 5 Department of Orthopedic Surgery, The Second Affiliated Hospital of Nanchang University, Nanchang, Jiangxi, China

**Keywords:** arthroscopic-assisted uniportal spinal surgery, lumbar degenerative disease, minimally invasive surgery, spinal endoscopy, two-segment lumbar disc herniation, unilateral biportal endoscopy, uniportal non-coaxial spinal endoscopic surgery

## Abstract

**Purpose:**

To compare the clinical efficacy and perioperative outcomes of arthroscopy-assisted uniportal spinal surgery (AUSS) and unilateral biportal endoscopy (UBE) for the treatment of adjacent two-segment lumbar disc herniation (AT-LDH).

**Methods:**

In this retrospective cohort study, we analyzed the data of 59 patients with AT-LDH admitted to our department between January 2020 and May 2025. Of these, 33 patients underwent AUSS and 26 underwent UBE. The primary outcome measures were the visual analog scale (VAS) for low back and leg pain and Oswestry disability index (ODI). Secondary outcomes included operative time, number of intraoperative fluoroscopies, incision length, length of hospital stay, perioperative hemoglobin change, complications, facet joint preservation, and serum inflammatory markers.

**Results:**

Both the AUSS and UBE groups exhibited significant postoperative improvements in VAS and ODI compared with baseline (*p* < 0.05). No significant between-group differences in clinical efficacy were observed at any time point (*p* > 0.05). The excellent-to-good rates were 87.9% for AUSS and 88.5% for UBE. No significant between-group differences in complication and facet joint preservation rates were found (AUSS: 6.1% vs. UBE: 7.7% and AUSS: 81.6% vs. UBE: 83.2%, respectively). However, the AUSS group showed shorter incision length and operative time, fewer fluoroscopic exposures, smaller postoperative hemoglobin decreases, and lower levels of inflammatory markers on postoperative day 3 (all *p* < 0.05).

**Conclusion:**

Both AUSS and UBE provided satisfactory short-term clinical outcomes for AT-LDH. AUSS offers advantages of reduced invasiveness, greater surgical efficiency, and lower inflammatory response, supporting its broader clinical application.

## Introduction

**Figure 1:**
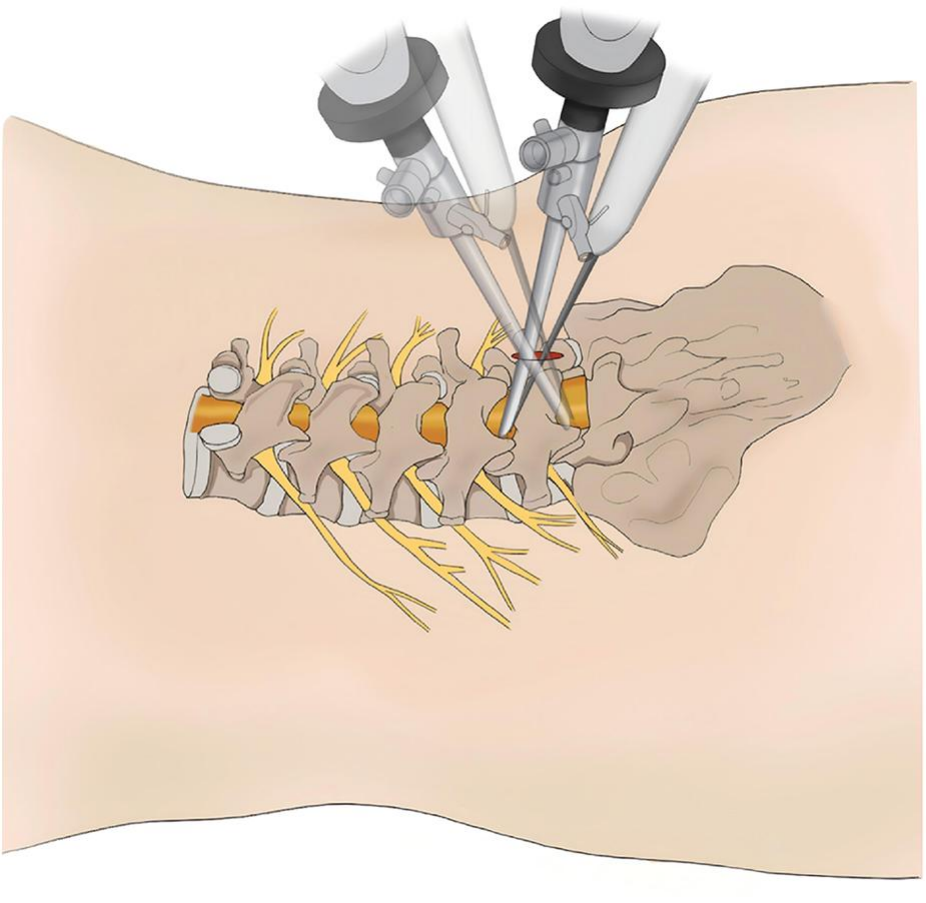
Schematic diagram illustrating the AUSS technique for AT-LDH discectomy performed through a single incision. AT-LDH, adjacent two-segment lumbar disc herniation; AUSS, arthroscopic-assisted uniportal spinal surgery.

Lumbar disc herniation (LDH) is a prevalent orthopedic condition characterized by lower back pain, leg pain, and neurological dysfunction (1). With global population aging and lifestyle changes, its incidence continues to rise and shows a trend toward earlier onset (2, 3). Surgical intervention is indicated when symptoms persist despite standard conservative treatment. Traditionally, open discectomy has been the standard surgical approach for symptomatic LDH. In recent years, advances in minimally invasive techniques and endoscopic instrumentation have driven the increasing adoption of endoscopic spinal surgery (4–6). Arthroscopy-assisted uniportal spinal surgery (AUSS), also known as uniportal non-coaxial spinal endoscopic surgery (UNSES), integrates the observation and operative channels within a single incision (7). By using standard spinal instruments, AUSS improves surgical efficiency while retaining the familiar workflow of open surgery, effectively functioning as an endoscopic laminotomy (8). This technique combines the advantages of non-coaxial endoscopy, such as a wide field of view and flexible maneuverability, with the multi-angle swinging and rotational capabilities typically associated with coaxial endoscopy, enabling versatile multi-angle operations through a single incision (9). Furthermore, wide-range swinging expands the surgical field, enabling single-incision discectomy for adjacent two-segment LDH (Figure 1). Favorable outcomes have been reported for AUSS in single-segment LDH (10). However, no clinical studies have evaluated its application in adjacent two-segment LDH (AT-LDH). Therefore, we aimed to compare the clinical efficacy and perioperative safety of AUSS and unilateral biportal endoscopy (UBE) for two-segment LDH, evaluate the clinical utility of AUSS in AT-LDH, and provide evidence to guide treatment selection.

## Methods

### Study design and patient selection

This retrospective cohort study was approved by the Ethics Committee of the 908th Hospital of the Joint Logistics Support Force (Approval No. 908YYLL20250035). A total of 59 patients with AT-LDH who underwent surgery in the Department of Orthopedics at our hospital between January 2020 and May 2025 were included. All patients had received standardized conservative treatment for at least 3 months but failed to respond.

Patients were grouped by surgical approach: AUSS (*n* = 33) and UBE (*n* = 26). Group allocation was determined chronologically based on the primary surgeon's evolving practice and introduction of the AUSS technique at our institution. Specifically, patients treated earlier in the study period (January 2020 to mid-2023) predominantly underwent UBE, whereas those treated later (mid-2023 to May 2025) predominantly underwent AUSS. A single experienced spinal surgeon proficient in both UBE and AUSS procedures performed all surgeries, thereby minimizing inter-operator variability. Demographic and baseline clinical information, including age, sex, body mass index (BMI), smoking status, comorbidities, disease duration, affected spinal segments, and follow-up duration, were collected (Table 1). Preoperative imaging assessments included lumbar radiography, computed tomography (CT), and magnetic resonance imaging (MRI).

**Table 1:** Baseline characteristics and perioperative indicators in the AUSS and UBE groups.

General data	AUSS (*n* = 33)	UBE (*n* = 26)	*p*-value
Age (years)	55.70 ± 11.91	52.08 ± 13.26	0.275
Sex, *n* (%)			0.522
Male	18 (54.5)	12 (46.2)	
Female	15 (45.5)	14 (53.8)	
BMI (kg/m²)	25.68 ± 2.56	24.88 ± 2.89	0.260
Smoking, *n* (%)	8 (24.2)	4 (15.4)	0.521
Comorbidities, *n* (%)			
Hypertension	11 (33.3)	7 (26.9)	0.595
Diabetes	9 (27.3)	4 (15.4)	0.351
Disease duration (months)	11.24 ± 6.69	10.15 ± 5.95	0.517
Lesion segments, *n* (%)			1.000
L3/4 and L4/5	4 (12.1)	3 (11.5)	
L4/5 and L5/S1	29 (87.9)	23 (88.5)	
Follow-up time (months)	12.94 ± 4.87	14.27 ± 7.10	0.397

Data are presented as mean ± standard deviation (SD) or number (%).

AUSS, arthroscopic-assisted uniportal spinal surgery; BMI, body mass index; UBE, unilateral biportal endoscopy.

### Inclusion and exclusion criteria

Patients with (1) low back pain, leg pain, or lower limb numbness or weakness; (2) MRI-confirmed AT-LDH consistent with clinical symptoms; (3) failure of ≥3 months of conservative treatment; and (4) ≥6 months of follow-up were included.

The exclusion criteria included (1) far-lateral disc herniation; (2) lumbar instability on imaging; (3) coexistent lumbar spinal stenosis; and (4) tumors, infections, or coagulation disorders.

### Surgical procedures

#### AUSS

**Figure 2:**
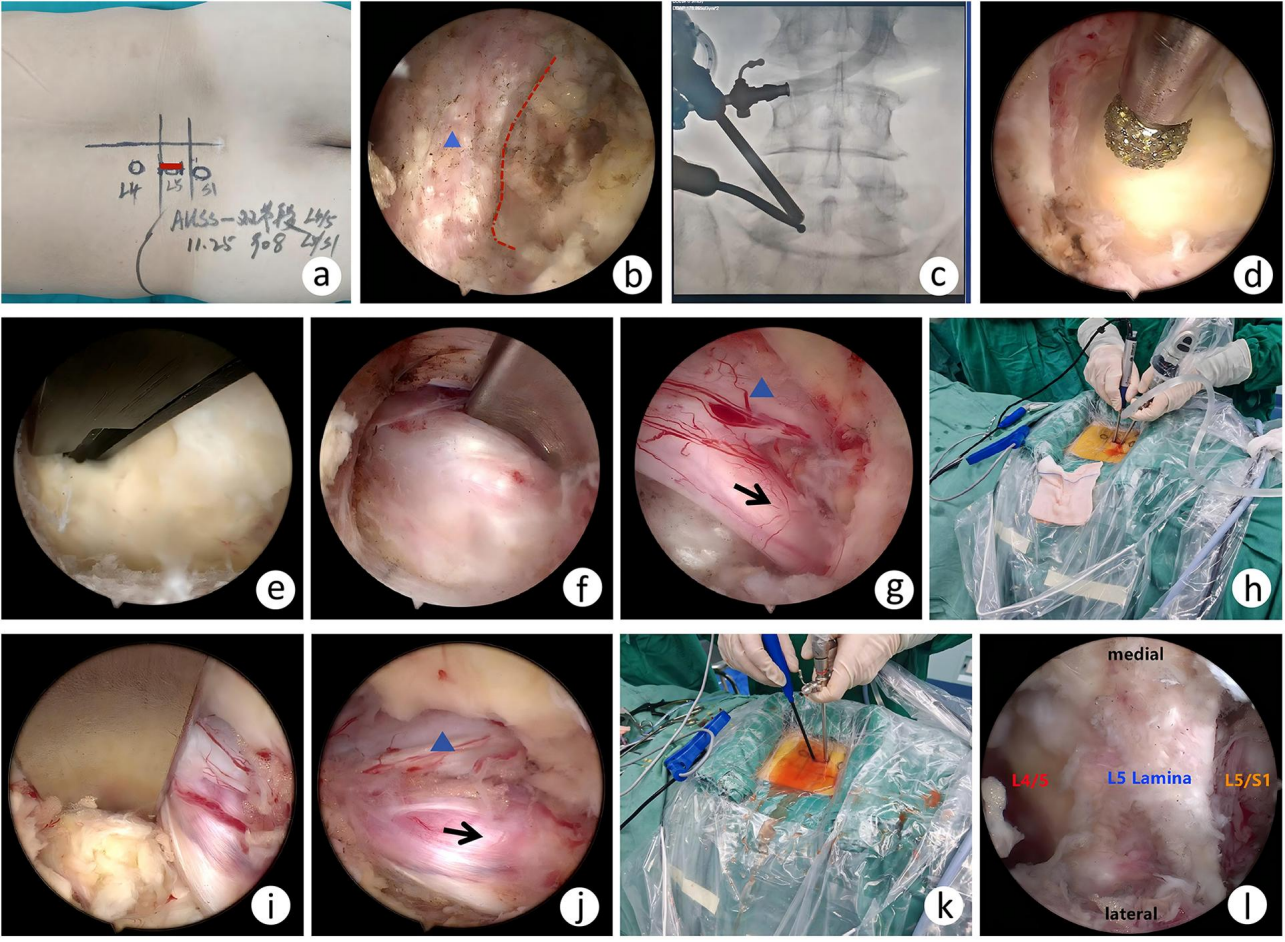
S**c**hematic diagram of the AUSS procedure. **(a)** Incision and surface marking of the affected segments. **(b)** Endoscopic landing point (dashed line: inferior border of the lamina of the superior vertebra; triangle: lamina). **(c)** Intraoperative fluoroscopy confirming the target segment. **(d)** Drilling the medial edge of the superior lamina and facet joint. **(e)** Resection of the ligamentum flavum. **(f)** Exposure of the L5/S1 herniated disc. **(g)** Decompression with S1 nerve root release (arrow: S1 traversing nerve root; triangle: dural sac). **(h)** Caudad tilting for endoscopic manipulation at L5/S1. **(i)** Exposure of the L4/L5 herniated disc. **(j)** Decompression with L5 nerve root release (arrow: L5 traversing nerve root; triangle: dural sac). **(k)** Cephalad tilting for endoscopic manipulation at L4/L5. **(l)** The interlaminar spaces of two segments are simultaneously visualized under endoscopy.

Under general anesthesia, patients were positioned in the prone position. For left-sided L4/5 and L5/S1 disc herniations, a 2-cm longitudinal incision was made at the medial border of the L5 pedicle. After incision of the lumbodorsal fascia (the extent of the fascial incision must exceed the skin incision width intraoperatively), the paraspinal muscles were bluntly dissected to expose the laminar space at the L5 spinous process–lamina junction. A 30° arthroscope (Stryker) and right-angled radiofrequency plasma knife (Fangrun Technology Co., Ltd.) were then inserted. The 3,000 mL normal saline bag was positioned 50 cm above the surgical site to maintain adequate hydrostatic pressure. Soft tissue was cleared to expose the bony landmarks, and the surgical level was confirmed by fluoroscopy. A 4-mm drill (Zirui Technology Co., Ltd.) was used to resect the inferior edge of the L5 lamina, medial portion of the inferior articular process, and superior edge of the S1 lamina up to the insertion of the ligamentum flavum. The ligamentum flavum was then removed using a laminectomy punch to expose the dural sac and nerve root. A radiofrequency electrode (Fangrun) was used to coagulate vessels near the nerve root and the vertebral venous plexus at the posterior edge of the vertebral body. The dural sac and traversing nerve root were retracted medially using a nerve retractor, and the herniated nucleus pulposus was removed with nucleus pulposus forceps. The nerve root was further explored both proximally and distally to ensure complete removal of residual disc material. Annular fibrosus repair was assessed based on the extent of damage. The arthroscope was then retracted and advanced caudally along the L5 lamina to the L4 spinous process–lamina junction, and the same procedure was repeated for the L4/5 level. Finally, a drainage tube was placed, and the incision was sutured (Figure 2).

#### UBE

For two-segment LDH, the order of UBE treatment varied according to the side of herniation: the distal segment was addressed first for left-sided herniations, whereas the proximal segment was treated first for right-sided herniations, ensuring clear vision and unobstructed irrigation flow. For example, in cases of left L4/5 and L5/S1 AT-LDH, the same anesthesia and patient positioning as those used in AUSS were applied. The C-arm localized the pedicle projections and midline. First, L5/S1 discectomy was performed: a 0.5-cm incision at the medial edge of the L5 pedicle served as the observation channel, and a 1.5-cm incision at the medial edge of the S1 pedicle served as the working channel. The lumbodorsal fascia was incised, and muscle was dissected with dilators to the L5 spinous process–lamina junction, confirmed by fluoroscopy. The 30° arthroscope (Stryker) and plasma radiofrequency knife were inserted, and a 3,000 mL normal saline bag was hung 50 cm higher than the level of the surgical site; subsequent steps were identical to AUSS. For L4/5 discectomy, the arthroscope was withdrawn, the L5 incision was extended to 1.5-cm as the working channel, and a 0.5-cm incision was made at the L4 pedicle as the observation channel. Dissection and channel placement were repeated, and endoscopic steps mirrored those above. A drainage tube was placed at the L5 incision, and the wound was sutured.

### Postoperative management

All patients received a single intravenous infusion of 1.5 g of cefuroxime sodium for postoperative infection prevention. Oral nonsteroidal anti-inflammatory drugs were prescribed as needed to relieve pain. Lumbar muscle exercises and straight leg raises were encouraged to prevent atrophy and nerve root adhesion. From postoperative day 3, patients ambulated with a lumbosacral brace while avoiding bending, strenuous lumbar activity, and weight-bearing.

### Observation indicators and efficacy evaluation

Clinical Outcomes including operative time, incision length, number of intraoperative fluoroscopies, preoperative/postoperative day 3 hemoglobin difference, hospital stay, and perioperative complications were recorded. Regarding pain assessment, VAS scores for low back and leg pain were evaluated preoperatively and at 3 days, 3 months, and 6 months postoperatively.

Lumbar function was assessed using the ODI (lower scores indicate better recovery). At 6 months, outcomes were graded using the modified MacNab criteria (excellent, good, fair, and poor), with the excellent–good rate calculated as follows: (excellent + good cases)/total cases × 100%. For radiographic measurements, facet joint preservation was assessed by measuring the width of facet joints on axial MRI slices before and after surgery, employing the method described by Pao (11). Additionally, serum C-reactive protein (CRP) and interleukin-6 (IL-6) levels, were measured preoperatively and on postoperative day 3 to evaluate the inflammatory response to surgical trauma.

### Statistical methods

Data were analyzed using IBM SPSS Statistics version 24.0 (IBM Corp., Armonk, NY, USA). Continuous variables are expressed as mean ± standard deviation (SD). When the data were normally distributed, the independent samples t-test was used for comparison between the two groups, and one-way analysis of variance was applied for intragroup comparisons among different time points. When the data were non-normally distributed, the rank-sum test was adopted. Categorical variables were compared using Fisher's exact test or chi-square test. The Mann–Whitney *U* test was used for ordinal data. A two-tailed *p*-value <0.05 was considered statistically significant.

## Results

### Baseline characteristics

All 59 patients underwent surgery and completed at least 6 months of follow-up. No significant differences were observed in baseline demographics between the AUSS and UBE groups (*p* > 0.05; Table 1). The groups were comparable in age, sex, BMI, smoking status, comorbidities, symptom duration, affected segments, and follow-up duration. Postoperative imaging confirmed adequate decompression without residual compression in either group.

### Perioperative indicators

Significant differences were observed in perioperative parameters (Table 2). The AUSS group had a shorter mean incision length (2.06 ± 0.17 vs. 2.94 ± 0.25 cm; *p* < 0.001) and operative time (84.79 ± 10.84 vs. 93.15 ± 14.05 min; *p* = 0.012). The AUSS group required fewer fluoroscopy exposures than the UBE group (2.24 ± 0.75 vs. 3.23 ± 0.59 times, respectively; *p* < 0.001). No significant difference was found in hospital stay (*p* > 0.05). However, hemoglobin reduction on postoperative day 3 was lower in the AUSS group (13.61 ± 4.39 g/L) than in the UBE group (16.65 ± 3.20 g/L; *p* = 0.004), suggesting reduced blood loss.

**Table 2:** Comparison of perioperative indicators between the two groups.

Perioperative indicators	AUSS (*n* = 33)	UBE (*n* = 26)	*t*/*x*^2^	*p* value
Operative time (min)	84.79 ± 10.84	93.15 ± 14.05	2.583	0.012
Incision length (cm)	2.06 ± 0.17	2.94 ± 0.25	16.244	<0.001
Fluoroscopy (times)	2.24 ± 0.75	3.23 ± 0.59	5.509	<0.001
Hb difference (g/L)	13.61 ± 4.39	16.65 ± 3.20	2.969	0.004
Hospital stay (days)	7.58 ± 1.84	8.04 ± 1.87	0.954	0.344
Complications (n, %)	2 (6.1)	2 (7.7)	—	1.000
Dural tear (n, %)	1 (3.0)	1 (3.8)		
Nerve root injury (n, %)	0 (0)	1 (3.8)		
Inferior articular process destruction (n, %)	1 (3.0)	0 (0)		

AUSS, arthroscopic-assisted uniportal spinal surgery; Hb, hemoglobin; UBE, unilateral biportal endoscopy.

No major complications occurred in either group. One patient in the UBE group experienced transient sensory hypoesthesia, which resolved within 3 weeks. In the AUSS group, one patient with severe convergence of the inferior articular process underwent unintended complete resection of the L3 inferior articular process to obtain sufficient operative space; however, the patient did not experience severe low back pain postoperatively or at the final follow-up. Dural tears occurred in one patient in each group, with tear lengths <4 mm. Intraoperative endoscopic evaluation confirmed intact arachnoid membranes, no fibrin sealant was used, and no dural repairs were required. Postoperatively, no cerebrospinal fluid leakage was observed. The overall incidence of complications did not differ significantly between groups (*p* > 0.05; Table 2).

### Observation and efficacy indicators

Regarding clinical indicators, both groups demonstrated significant reductions in VAS and ODI scores at all follow-up time points (*p* < 0.05; Table 3), indicating symptomatic and functional improvement. However, no significant intergroup differences were observed (*p* > 0.05), suggesting comparable clinical efficacy. Considering the small sample size, type II errors are likely to occur. We conducted a *post hoc* power calculation for VAS and ODI between groups. The results showed that the *post hoc* power for VAS was 0.58 and that for ODI was 0.51, both indicating moderate power. At 6 months, 87.9% of patients in the AUSS group and 88.5% in the UBE group achieved excellent or good outcomes. The between-group differences were not statistically significant (*p* > 0.05; Table 4).

**Table 3:** Comparison of VAS scores for leg pain, low back pain, and ODI between the two groups.

Efficacy indicators	AUSS (*n* = 33)	UBE (*n* = 26)	*T* value	*p* value
Leg pain VAS				
Preoperative	7.12 ± 1.08	6.96 ± 1.08	0.564	0.575
3 days postop	3.12 ± 1.05*	3.15 ± 1.00*	0.120	0.905
3 months postop	2.18 ± 1.01*	2.19 ± 0.94*	0.041	0.968
6 months postop	1.21 ± 0.78*	1.15 ± 0.78*	0.284	0.777
Low back pain VAS				
Preoperative	4.70 ± 1.49	4.35 ± 1.72	0.839	0.405
3 days postop	2.79 ± 0.93*	2.81 ± 1.13*	0.074	0.941
3 months postop	1.39 ± 0.86*	1.54 ± 0.91*	0.625	0.535
6 months postop	0.76 ± 0.79*	0.88 ± 0.82*	0.604	0.548
ODI				
Preoperative	66.09 ± 8.69	65.00 ± 7.69	0.503	0.617
3 days postop	32.03 ± 6.29*	31.15 ± 6.82*	0.512	0.610
3 months postop	18.88 ± 5.64*	19.08 ± 5.46*	0.136	0.892
6 months postop	11.36 ± 4.40*	11.69 ± 4.96*	0.269	0.789

AUSS, arthroscopic-assisted uniportal spinal surgery; ODI, Oswestry disability index; VAS, visual analog scale; UBE, unilateral biportal endoscopy.

* *P* *<* 0.05 compared with preoperative values.

**Table 4:** Comparison of excellent–good rates between the two groups.

Group	Efficacy evaluated using MacNab criteria				
	Excellent	Good	Fair	Poor	Excellent–good rate
AUSS (*n* = 33)	19	10	4	0	87.9%
UBE (*n* = 26)	15	8	2	1	88.5%
*Z-*value					0.009
*p-*value					0.993

AUSS, arthroscopic-assisted uniportal spinal surgery; UBE, unilateral biportal endoscopy.

**Figure 3:**
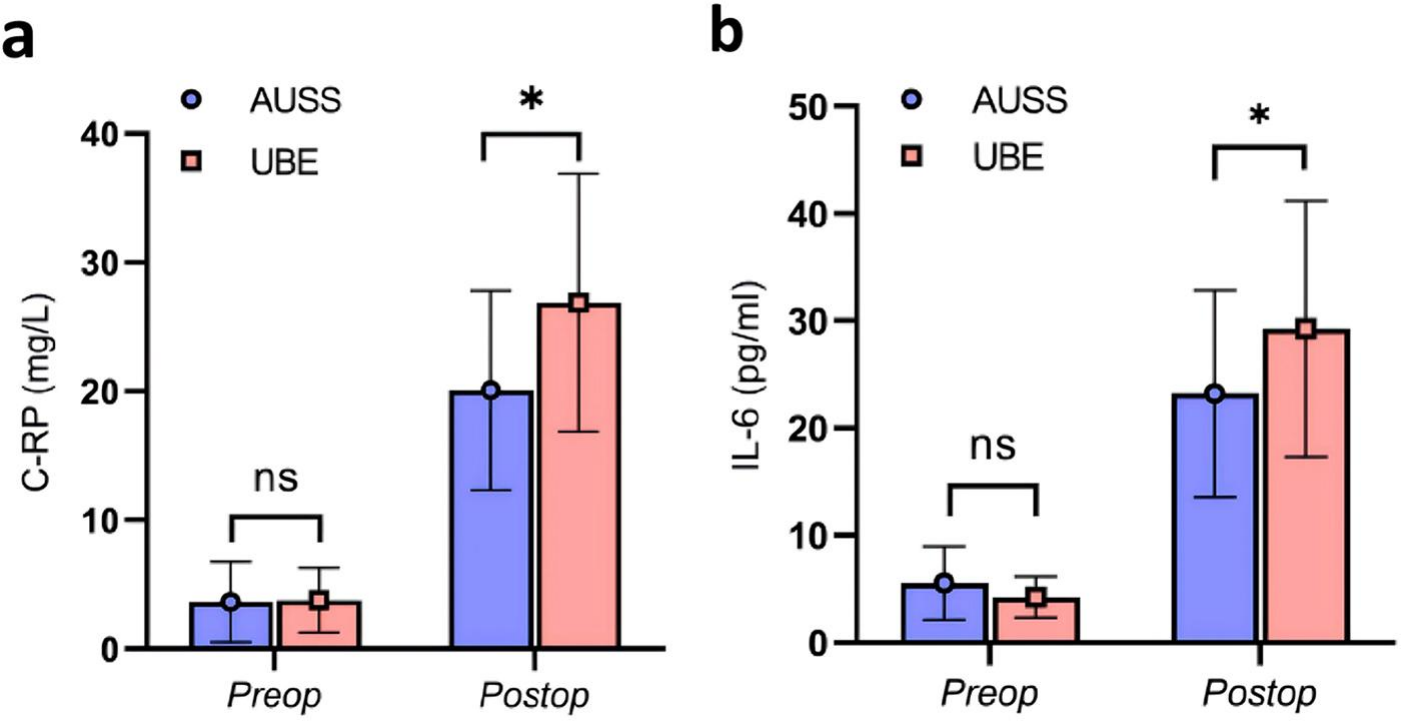
Inflammatory marker expression in the AUSS and UBE groups before and 3 days after surgery. CRP **(a)** and IL-6 **(b)** were significantly lower in the AUSS group than in the UBE group on postoperative day 3 (*p < 0.05 for both).

**Figure 4:**
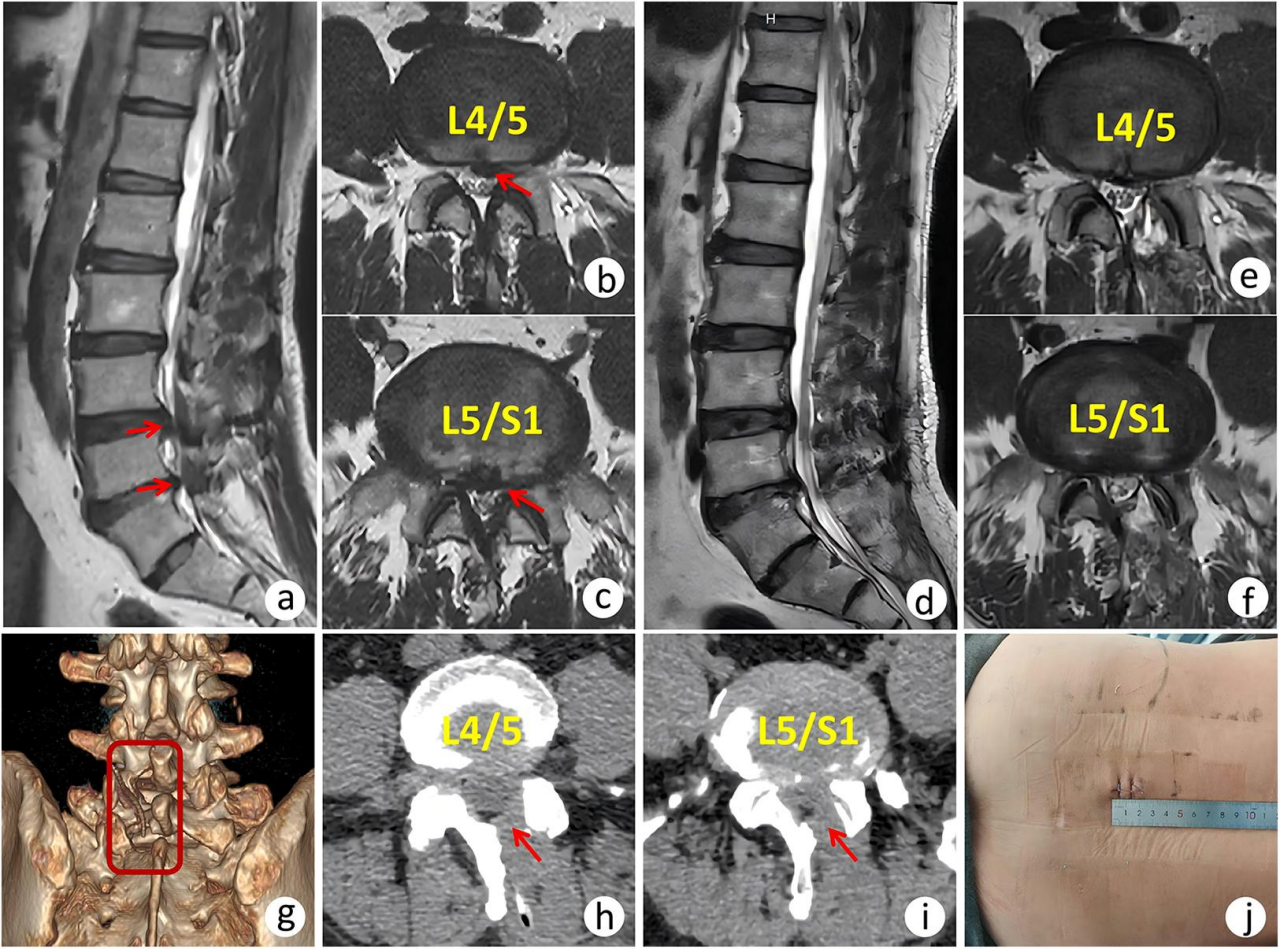
A 54-year-old male patient with L4/5 and L5/S1 AT-LDH treated by AUSS. **(a–c)** Preoperative MRI demonstrating disc herniation at L4/5 and L5/S1 levels (arrow). **(d–f)** Postoperative MRI demonstrating complete removal of the herniated disc. **(g)** Postoperative 3D reconstruction demonstrating partial laminectomy at L4 and L5 levels (boxed area). **(h–i)** Axial CT scan demonstrating laminar fenestration (arrow). **(j)** Postoperative incision length.

**Figure 5:**
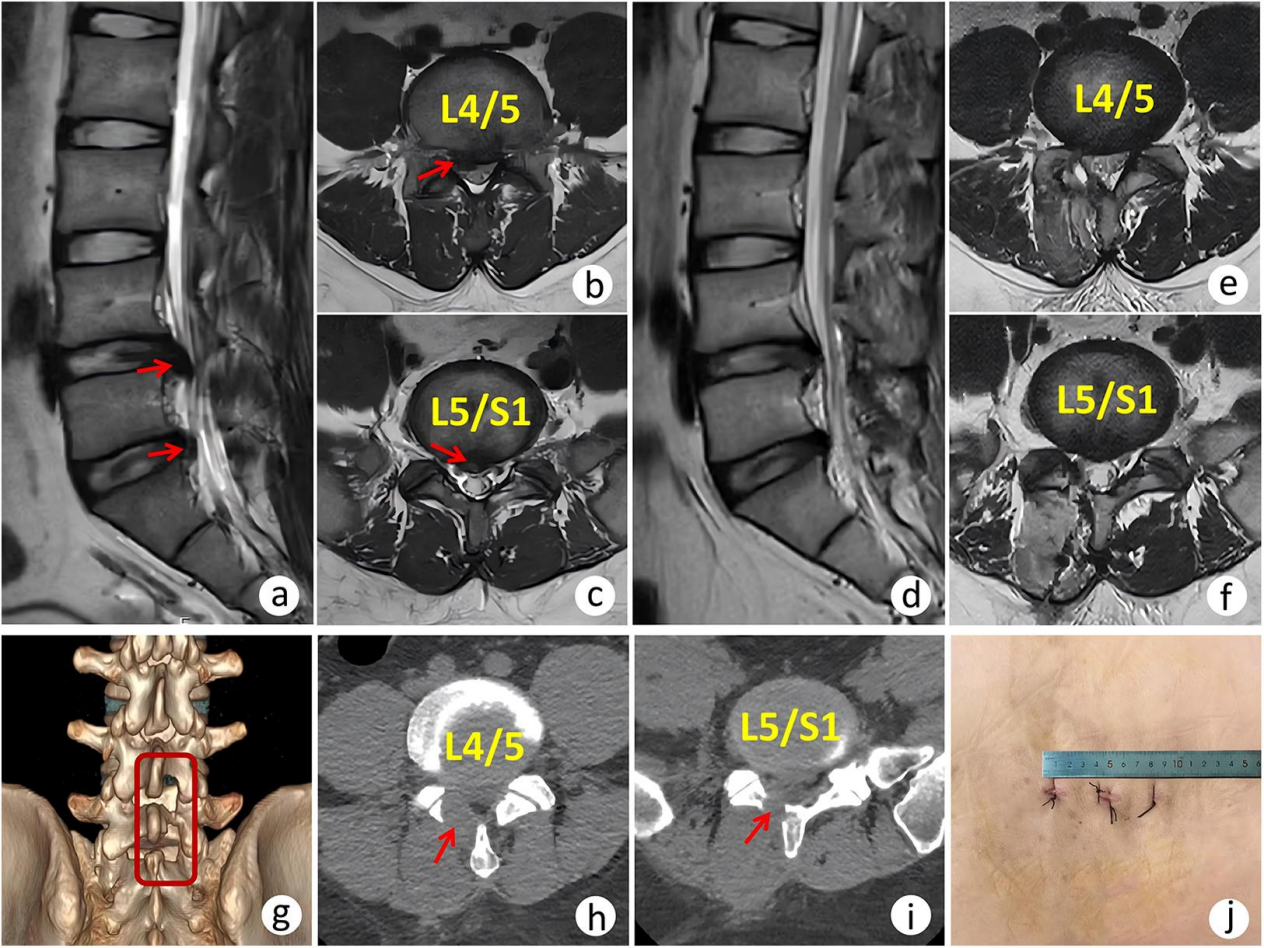
A 35-year-old male patient with L4/5 and L5/S1 AT-LDH treated by UBE. **(a–c)** Preoperative MRI demonstrating disc herniation at L4/5 and L5/S1 levels (arrow). **(d–f)** Postoperative MRI demonstrating complete removal of the herniated disc. **(g)** Postoperative 3D reconstruction demonstrating partial laminectomy at L4 and L5 levels (boxed area). **(h–i)** Axial CT scan demonstrating laminar fenestration (arrow). **(j)** Postoperative incision length.

Regarding radiographic Measurements, the facet joint preservation rate was 81.63% ± 9.95% in the AUSS group and 83.19% ± 5.57% in the UBE group. The between-group difference was not statistically significant (*p* = 0.314). For inflammatory markers, baseline serum CRP and IL-6 levels were comparable (*p* = 0.859 and *p* = 0.084, respectively). By postoperative day 3, both markers were significantly lower in the AUSS group than in the UBE group (*p* < 0.05), indicating a milder postoperative inflammatory response (Figure 3). A representative case of AUSS is shown in Figure 4, and a representative case of UBE is shown in Figure 5.

## Discussion

Multilevel LDH (MLDH) refers to herniation at two or more lumbar levels, compressing nerve roots or the spinal cord and causing low back and radiating leg pain. It predominantly affects middle-aged and older adults, with pathogenesis closely associated with chronic strain and disc degeneration (12). In contrast, onset in younger patients may be influenced by genetic factors, trauma, or occupational exposure. One study reported that 18% of young computer workers experienced low back pain due to MLDH, often involving multiple consecutive segments (13). Because of overlapping innervation and multilevel pathology, identifying the symptomatic level and planning surgery remain challenging (14). MRI, the primary imaging modality for LDH, can delineate disc morphology, herniation location, and neural compression (15, 16). Typically, the symptomatic level can be determined by correlating clinical symptoms, physical findings, and imaging findings. When findings are ambiguous or inconsistent, selective nerve root blocks (SNRBs) may assist in diagnosis (17). In this study, SNRBs confirmed both L4/5 and L5/S1 as symptomatic levels in six patients with unclear localization; two-level discectomy effectively relieved their pain.

AUSS integrates the operative and observational channels within a single surgical incision, achieving a high degree of coordination between the viewing and operating directions. Similar to the UBE technique, AUSS allows the direct use of traditional spinal instruments, aligning with the workflow of open surgery. It is essentially an endoscopic implementation of open spinal surgery, thereby lowering the learning curve for spinal endoscopic procedures. Surgeons with experience in open surgery can quickly master this technique (8). Currently, AUSS has been successfully applied in the treatment of various spinal diseases, such as LDH, lumbar spinal stenosis, and epidural lipomatosis (18–21). Moreover, through operative techniques such as coaxial swinging, coaxial rotation, non-coaxial crossing, and coaxial hand-switching, AUSS enables multidirectional and more flexible endoscopic manipulation. This characteristic broadens the scope of endoscopic decompression and enables surgical treatment for AT-LDH through a single incision.

This study retrospectively analyzed the clinical outcomes of AUSS and UBE in treating AT-LDH. The excellent–good rates were 87.9% for AUSS and 88.5% for UBE. At 6 months postoperatively, both groups showed significant improvements in low back and leg pain VAS and ODI scores from baseline. Postoperative imaging confirmed complete resection of herniated nuclei and adequate neural decompression, indicating that both techniques effectively achieve two-segment discectomy, relieve pain, and improve lumbar function. However, AUSS was superior in incision length, surgical procedure, operative time, and number of fluoroscopies. These advantages likely result from several factors. First, UBE requires at least three incisions for two-segment surgery, whereas AUSS, using coaxial swing, completes the procedure through a single incision, reducing the number of incisions and surgical trauma and lowering the risk of postoperative complications such as infection. Second, when treating the second-segment herniation, AUSS avoids arthroscope withdrawal, re-establishing the working channel, or repeated fluoroscopic localization. Instead, minor adjustments to the endoscope's tilt and advancement along the lamina suffice to access the target laminar space of the lower segment, shortening the operative time and reducing radiation exposure for both patients and surgeons. Third, in UBE, surgical field clarity depends on unobstructed water flow within the working channel; operative instruments often impede fluid egress, compromising visualization and efficiency. In contrast, AUSS preserves a gap between its observation channel and instruments, enabling uninterrupted water flow and consistent visual clarity. Fourth, UBE's “rendezvous” and “triangular” techniques require advanced hand– eye coordination and spatial cognition. Inexperienced surgeons may initially struggle to locate the plasma radiofrequency electrode for endoscopic rendezvous due to limited familiarity with triangular positioning, reducing efficiency (22). In contrast, AUSS integrates observation and working channels through a single incision, simplifying rendezvous and cavity creation and improving procedural efficiency.

In addition, the postoperative hemoglobin drop in the AUSS group was significantly less than that in the UBE group (13.61 ± 4.39 vs. 16.65 ± 3.20 g/L; *p* < 0.05), suggesting that AUSS provides better bleeding control. This advantage is attributed to fewer incisions and the ability of plasma radiofrequency electrode to promptly and precisely coagulate bleeding points in muscles during channel establishment, thereby reducing intraoperative and postoperative hidden blood loss. In this study, the CRP and IL-6 levels on postoperative day 3 were significantly lower in the AUSS group than in the UBE group (*p* < 0.05), indicating a milder inflammatory response with AUSS. The coaxial single-incision approach of AUSS minimizes tissue disruption, attenuates the surgical stress response, and lowers postoperative inflammatory marker levels.

Although AUSS technology demonstrates promising clinical prospects, its technical operation still presents certain challenges. In a 2-cm narrow incision, avoiding mutual interference between the endoscope and operating instruments is the primary problem that novice surgeons face when learning the AUSS technique. When dealing with bone tissue on the right side of the operator, the tail of the laminar rongeur may easily collide with the endoscope owing to limited channel space and operating angle of the instruments, hindering bone removal. Our experience is to take advantage of the coaxial swinging and rotating characteristics of AUSS. By rotating the laminar rongeur parallel to the arthroscope toward the left and placing the targeted area in front of or behind the laminar rongeur, the problem of instrument interference can be effectively addressed. In addition, during the operation, the orientation of the 30° endoscope needs to be dynamically adjusted according to different exposure sites to optimize the direction of the visual field. This provides an optimal endoscopic view and creates more space for endoscopic operations. Finally, choosing longer operating instruments (such as a 300-mm laminar rongeur) can increase the operating lever arm, keep the handle of the instrument away from the endoscope, and reduce the likelihood of collision. The combined application of these techniques can effectively improve procedural coordination.

Complication rates were comparable, and no major adverse events occurred, indicating that both techniques were safe. In the UBE group, one patient experienced lower-limb hypoesthesia due to inadvertent nerve root injury during the removal of axillary protruding nucleus pulposus tissue in a narrow space. To avoid this, instruments should be used only after clearly identifying the tissue structures, and gentle manipulation with minimal nerve traction is essential. Previous studies have also reported dural tears as common complications of non-coaxial endoscopic surgery (23–25). In this study, one dural tear occurred in each group; however, neither resulted in cerebrospinal fluid leakage, likely due to their small size and intact arachnoid membranes. Although conventional instruments improve efficiency, they may increase the risk of dural or nerve root injury. Therefore, when using a laminectomy punch or nucleus pulposus forceps to remove the ligamentum flavum, a nerve dissector should first be used to fully separate the ligamentum flavum from the dura. Progressive, delicate techniques are recommended to avoid tearing and reduce complication risk (26). In the AUSS group, one patient underwent complete resection of the inferior articular process. Although severe low back pain was not reported at the final follow-up, excessive removal of the articular process theoretically increases the risk of segmental instability, which could potentially lead to low back pain (27, 28). Therefore, preoperative CT-based planning to determine the extent of bone removal is essential for preserving facet joint integrity. Based on our practical experience, when using a 4-mm drill for vertebral lamina resection, it is essential to refer to the preoperative design to determine the scope of grinding. We have found it useful to treat the drill as a measuring tool. Generally, we take the junction between the base of the spinous process and the vertebral lamina as the medial starting point. The grinding range should not exceed three drill widths toward the lateral side. This approach helps in controlling the extent of bone removal more precisely and reduce the risk of excessive damage to the facet joint and other important structures, thereby minimizing the potential for iatrogenic instability. Wang et al. (29) reported that the surface width of the laminae of the upper lumbar vertebrae (L1, L2, and L3) was significantly smaller than that of the lower lumbar vertebrae (L4 and L5). Specifically, the safe laminectomy distances for L1, L2, and L3 were 7.75 ± 1.27, 8.60 ± 1.32, and 9.17 ± 1.18 mm, respectively. Consequently, for patients with high-level lumbar disc herniation, if preoperative assessment predicts that laminectomy may damage the facet joints, percutaneous transforaminal endoscopic discectomy could be a preferable surgical option.

However, a major limitation of this study is the relatively short minimum follow-up period. This restricts our ability to adequately assess medium-term outcomes, recurrence rates, and the development of adjacent segment disease. Another limitation is the temporal discrepancy in case enrollment between the two groups, which may introduce time-trend bias and selection bias. Furthermore, the limited sample size undermines the robustness of efficacy evaluation and increases the likelihood of type II error, potentially yielding false-negative findings. Although the study preliminarily verified the clinical efficacy of both surgical methods and the technical benefits of AUSS, large-sample, multicenter prospective randomized controlled studies with extended follow-up are needed to provide higher-level evidence for clinical decision-making.

In summary, both AUSS and UBE demonstrate favorable short-term clinical efficacy and safety profiles in treating AT-LDH. AUSS exhibits advantages in minimizing surgical trauma, reducing operative time, and controlling intraoperative blood loss, establishing it as a promising alternative for the minimally invasive treatment of AT-LDH. However, caution should be exercised to avoid compromising the facet joint during bony window creation.

## Data Availability

The raw data supporting the conclusions of this article will be made available by the authors, without undue reservation.
